# Morpho-molecular characterization of Colombian and Brazilian populations of *Rotylenchulus* associated with *Musa* spp

**DOI:** 10.21307/jofnem-2019-047

**Published:** 2019-08-05

**Authors:** Donald Riascos-Ortiz, Ana Teresa Mosquera-Espinosa, Francia Varón De Agudelo, Claudio Marcelo Gonçalves de Oliveira, Jaime Eduardo Muñoz-Flórez

**Affiliations:** 1 Facultad de Ciencias Agropecuarias, Universidad Nacional de Colombia, Palmira, Colombia; 2 Departamento de Ciencias Naturales y Matemáticas, Pontificia Universidad Javeriana, Cali, Colombia; 3 Centro de Investigación en Palma de Aceite-Cenipalma, Bogotá, Colombia; 4 Laboratorio de Nematologia, Instituto Biológico, Campinas, São Paulo, Brazil

**Keywords:** Banana, Diagnosis, Phytonematodes, Plantain, *Rotylenchulus reniformis*, Taxonomy

## Abstract

Three populations, two from Colombia and one from Brazil, of *Rotylenchulus reniformis* associated with banana and plantain, were characterized using morphological, morphometric, and molecular methods. Morphometric data from these populations were similar to type and reference populations of *R. reniformis*. Partial sequences of both D2-D3 rDNA and mitochondrial cytochrome oxidase subunit I (COI) regions had a strong affinity (99% similarity) to previously published sequences of *R. reniformis*. Phylogenetic analyses (maximum likelihood and Bayesian inference) suggested that the Colombian populations of *R. reniformis* corresponded to the previously described Type A of the species. This is the definitive first report in Colombia of *R. reniformis* associated with banana and plantain crops.

Colombia is the fourth largest global producer of plantain (*Musa* spp.) after Uganda, Cameroon, and Ghana, with a production of 3,575,706 t in 2017. Recently, the production of plantain has increased in Colombia by 11% since 2012 with a current yield of 8.1 t ha^−1^ (FAO, 2018). Regional variation in yield of both banana and plantain exists, ranging from 35.9 t ha^−1^ yr^−1^ (Tolima municipality) to 107.8 t ha^−1^ yr^−1^ (Valle del Cauca) and from 274.2 t ha^−1^ yr^−1^ (Meta) to 542.1 t ha^−1^ yr^−1^ (Arauca) for banana and plantain, respectively (MADER, 2018).

Second only to black sigatoka disease caused by *Mycosphaerella fijiensis* Morelet (Araya, 2003), phytonematodes are considered the most limiting factor of *Musa* spp production in Colombia. The most destructive phytonematodes associated with *Musa* spp. are known to be *Radopholus similis* (Cobb, 1983; Thorne, 1949), *Pratylenchus* spp., *Meloidogyne incognita* (Chitwood, 1949), *M. javanica* (Chitwood, 1949), *Helicotylenchus multicinctus* (Cobb, 1893; Golden, 1956), *H. dihystera* (Cobb, 1893; Sher, 1961), *H. erythrinae* (Zimmermann, 1904), and *Rotylenchulus reniformis* (Linford and Oliveira, 1940; Ravichandra, 2014).

Of those, *Rotylenchulus* is a semi-endoparasitic and sedentary phytonematode that is of economic importance throughout (sub-)tropical and temperate zones reducing yield and quality of almost 150 crop species (Castillo and Gómez-Barcina, 1993; Robinson et al., 1997; Crozzoli et al., 2004; Khan, 2005; Moore and Lawrence, 2012; Jones et al., 2013). The genus *Rotylenchulus* currently comprises 11 valid species: *R. borealis*, *R. clavicaudatus*, *R. eximius*, *R. leptus*, *R. macrodoratus*, *R. macrosoma*, *R. macrosomoides*, *R. parvus*, *R. reniformis*, *R. sacchari*, and *R. vitis* (Germani, 1978; Robinson et al., 1997; Van den Berg et al., 2015; Palomares-Rius et al., 2018) with two species *R. borealis* and *R. reniformis* previously reported to be associated with *Musa* spp. (Van den Berg et al., 2003; Gaidashova et al., 2004; Jones et al., 2013; Daneel et al., 2015). *R. reniformis* was first reported to be associated with *Musa* spp. from Puerto Rico (Ayala and Roman, 1963) and later from Ivory Coast (Fargette and Quénéhervé, 1988), Brazil (Costa Manso et al., 1994), Vietnam (Ngoc Chau et al., 1997), India (Khan and Hasan, 2010), Democratic Republic of Congo (Kamira et al., 2013), and South Africa (Daneel et al., 2015). The species of Musaceae affected by *R. reniformis* include *M. acuminata* Colla, *M. balbisiana* Colla, *M. cavendishii* Lamb. Ex Paxton, *Musa martini* Hort. *ex* Carriere, *Musa paradisiaca* L., and *M. sapientum* L. (Robinson et al., 1997; Khan, 2005). Symptoms and damage in *Musa* spp. attributed to *R. reniformis* include necrosis and reduction of secondary root development, stunting, chlorosis of aerial vegetation, and restricted development and reduced yield of banana and plantain. Significant yield losses of between 25 and 60% have been recorded with population levels of 0.1 to 10 *R. reniformis* cm^3^ of soil (Robinson et al., 1997, Crozzoli et al., 2004; Jones et al., 2013).

While numerous reports of *Rotylenchulus* associated with *Musa* spp. in Colombia exist (Zuñiga et al., 1979; Barriga and Cubillos, 1980; Curiel and Ospino, 2001; Gómez, 2001; Guzmán et al., 2012), detailed morphological, morphometric, and molecular data were not included. Thus, in Colombia, there is limited knowledge as to which species of *Rotylenchulus* are associated with *Musa* spp. which impedes the deployment of effective management strategies to control the species. To address this knowledge gap, the present study aims to: identify by morphological, morphometric, and molecular analysis the species of *Rotylenchulu*s associated with *Musa* spp. in Colombia, and analyze the phylogenetic relationship of *Rotylenchulus* species.

## Materials and methods

### Sampling, extraction, morphological, and morphometric analyses of nematodes

Soil and root samples of banana and plantain were collected from farms in Bolo and Rozo (Palmira, Valle del Cauca, Colombia) and Minas Gerais (Brazil) between 2016 and 2018. Composite soil and root samples of 1 kg were collected from each sampled farm from the root zone of 15 to 20 randomly selected plants ha^−1^. Secondary and tertiary roots and soil were collected to a distance of 25 cm of the pseudostem and among 0 to 30 cm of profundity with aid of a spade, soil auger and knife. A modification of Cobb’s method was used to extract the nematodes from soil and root (Ravichandra, 2014). Nematodes were killed by heat at 65°C for 4 min and then fixed with 2% formalin (Rosa et al., 2014). Key morphometric measurements for the genus (Table 1) were taken according to Robinson et al. (1997), Van den Berg et al. (2015), and Palomares-Rius et al. (2018). Microphotographs were taken using a light microscope equipped with differential interference contrast-DIC (DM2500, Leica, Germany).

**Table 1.** Morphometric data of studied populations for *R. reniformis*.

	*Rotylenchulus reniformis*		*Rotylenchulus reniformis*		*Rotylenchulus reniformis*	
Locality/crop	Bolo – Palmira (Valle del Cauca)-Banana*		Rozo – Palmira (Valle del Cauca)-Plantain*		Minas Gerais – Brasil Banana*	
Sex	Immature female	Male	Immature female	Male	Immature female	Male
	*n* = 15	*n* = 7	*n* = 5	*n* = 4	*n* = 20	*n* = 5
L	367.2 ± 23.8 (345.0–425.0)	366.6 ± 23.6 (335.0 –399.0)	367.5 ± 23.7 (332.5–390.0)	384.4 ± 23.1 (350.0–400.0)	392.0 ± 26.3 (342.5–430.0)	385.5 ± 34.8 (327.5–415.0)
a	23.8 ± 1.1 (21.8–26.6)	25.9 ± 1.1 (23.9–27.3)	23.0 ± 0.9 (22.2–24.4)	27.1 ± 2.3 (25.0–30.4)	24.8 ± 1.5 (21.9–27.0)	27.1 ± 2.3 (23.4–29.6)
b’	2.9 ± 0.2 (2.5–3.1)	3.9 ± 0.2 (3.4–4.1)	2.9 ± 0.2 (2.6–3.2)	3.8 ± 0.1 (3.7–4.0)	3.0 ± 0.2 (2.6–3.4)	3.8 ± 0.1 (3.6–3.9)
c	16.1 ± 1.1 (14.1–17.6)	16.8 ± 1.2 (15.3–19.1)	17.3 ± 1.9 (15.0–19.6)	17.5 ± 0.5 (17.1–18.2)	16.6 ± 1.2 (14.2–19.0)	18.4 ± 2.2 (15.6–21.4)
c’	2.3 ± 0.3 (2.0–2.8)	2.1 ± 0.2 (1.8–2.5)	2.1 ± 0.4 (1.7–2.6)	1.9 ± 0.3 (1.7–2.3)	2.4 ± 0.2 (2.0–2.9)	1.8 ± 0.3 (1.5–2.3)
DGO	16.4 ± 2.0 (13.0–20.0)	–	17.0 ± 1.0 (16.0–18.0)	–	17.2 ± 1.2 (15.0–19.0)	–
V or T	72.0 ± 0.9 (71.0–73.8)	–	72.2 ± 1.4 (70.9–74.4)	–	71.7 ± 1.1 (69.5–73.7)	–
Stylet length	16.0 ± 0.6 (15.0–17.0)	10.5 ± 0.6 (10.0–11.0)	15.4 ± 0.5 (15.0–16.0)	11.0 ± 0.8 (10.0–12.0)	15.8 ± 0.5 (15.0–17.0)	10.6 ± 0.5 (10.0–11.0)
Pharynx length	128.5 ± 8.3 (114.0–142.0)	95.0 ± 6.1 (85.0–103.0)	126.8 ± 8.7 (116.0–136.0)	100.3 ± 4.3 (95.0–105.0)	131.8 ± 9.5 (110.0–150.0)	100.2 ± 6.7 (90.0–108.0)
Excretory pore	77.7 ± 4.0 (72.0–84.0)	70.7 ± 4.1 (65.0–74.0)	81.0 ± 6.3 (73.0–89.0)	73.0 ± 2.4 (70.0–76.0)	79.9 ± 4.3 (72.0–88.0)	71.8 ± 4.0 (66.0–77.0)
Maximum body diam.	15.9 ± 1.2 (15.0–19.0)	14.1 ± 0.7 (13.0–15.0)	16.0 ± 0.7 (15.0–17.0)	14.3 ± 1.0 (13.0–15.0)	15.8 ± 0.5 (15.0–17.0)	14.2 ± 0.4 (14.0–15.0)
Anal body diam.	9.9 ± 1.1 (8.0–12.0)	10.3 ± 0.5 (10.0–11.0)	10.3 ± 0.5 (10.0–11.0)	11.8 ± 1.3 (10.0–13.0)	10.2 ± 0.5 (9.0–11.0)	11.6 ± 1.1 (10.0–13.0)
Lip region height	3.3 ± 0.5 (3.0–4.0)	6.1 ± 0.4 (6.0–7.0)	3.4 ± 0.5 (3.0–4.0)	3.5 ± 0.6 (3.0–4.0)	3.3 ± 0.5 (3.0–4.0)	3.2 ± 0.4 (3.0–4.0)
Lip region width	7.5 ± 0.6 (7.0–9.0)	3.6 ± 0.8 (3.0–5.0)	7.8 ± 0.4 (7.0–8.0)	6.0 ± 0.0 (6.0–6.0)	7.3 ± 0.5 (7.0–8.0)	6.0 ± 0.0 (6.0–6.0)
Tail length	22.8 ± 2.3 (20.0–27.0)	21.9 ± 1.9 (20.0–25.0)	21.8 ± 3.8 (17.0–26.0)	22.0 ± 1.4 (20.0–23.0)	23.7 ± 1.9 (21.0–27.0)	21.2 ± 3.6 (17.0–25.0)
h	7.3 ± 1.4 (5.0–11.0)	6.9 ± 1.2 (6.0–9.0)	8.3 ± 2.1 (6.0–11.0)	6.5 ± 1.3 (5.0–8.0)	7.1 ± 1.2 (5.0–10.0)	6.6 ± 1.5 (5.0–9.0)
Spicule length	–	17.4 ± 2.1 (15.0–21.0)	–	20.8 ± 1.0 (20.0–22.0)	–	20.6 ± 2.1 (18.0–23.0)
Gubernaculum length	–	6.9 ± 0.7 (6.0–8.0)	–	7.3 ± 1.3 (6.0–9.0)	–	7.3 ± 1.0 (6.0–8.0)

Notes: L, total body length; a, total body length divided maximum body diameter; b’, total body length divided by distance from anterior end of body to posterior end of pharyngeal glands; c, total body length divided by tail length; c’, tail length divided by body diameter at the anal/cloacal aperture; DGO, dorsal esophageal gland orifice; V or T, position of vulva or testis from anterior end expressed as percentage of body length; h, tail hyaline length. *Measurements in μm; mean ± SD (range).

### Statistical analysis

Morphometric data generated from this study and data sourced from the literature for other *Rotylenchulus* species (Van den Berg et al., 2003, 2015; Agudelo et al., 2005) were subjected to principal component analysis (PCA) using Community Analysis Package (PISCES Conservation Ltd, Lymington, UK) (Henderson and Seaby, 2014).

### Molecular analysis

Nematode DNA extraction followed Múnera et al. (2009) with modifications. A single nematode was crushed with a sterile scalpel and transferred to an Eppendorf tube with 15 µl worm lysis buffer (50 mM KCl, 10 Mm Tris pH 8.0, 15 mM MgCl2, 0.5% Triton x-100, 4.5% Tween-20, 0.09% Proteinase K). Subsequently, the tube was stored at −80°C for 15 min, incubated at 65°C for 1 hr and thereafter at 95°C for 15 min. Finally, the tube was centrifuged at 16,000 g for 1 min and stored at −20°C until further processing. The D2-D3 expansion region of the large subunit (LSU) of ribosomal DNA (28 S) was amplified using primers D2A (forward, 5′-ACAAGTACCGTGAGGGAAAGTTG-3′) and D3B (reverse, 5′-TCCTCGGAAGGAACCAGCTACTA-3′) (De Ley et al., 1999). Also, a partial region of the mitochondrial cytochrome oxidase subunit I (COI) was amplified using primers JB3 (forward, 5′-TTTTTTGGGCATCCTGAGGTTTAT-3′) and JB4.5 (reverse, 5′-TAAAGAAAGAACATAATGAAAATG-3′) (Bowles et al., 1992). The PCR conditions were initial denaturation during 2 min at 94 ºC followed by 40 cycles of 45 sec at 94 °C, 45 sec at 55 °C, 1 min at 72 °C and final extension of 10 min at 72 ºC for the amplification of D2-D3; initial denaturation during 2 min at 94 ºC followed by 40 cycles of 45 sec at 94 °C, 45 sec at 54 °C, 1 min at 72°C and final extension of 10 min at 72 ºC for the amplification of COI. PCR products were sequenced in both directions by BIONEER Korea.

### Phylogenetic analysis

Basic local alignment search tool (BLAST) at National Center for Biotechnology Information (NCBI) was used to confirm the species identity of the DNA sequences obtained in this study (Altschul et al., 1990). Consensus sequences were edited using Geneious software R6 (Biomatters; www.geneious.com) with multiple alignments performed in MAFFT v7 (Katoh et al., 2002) using sequences generated in this study and *Rotylenchulus* sequences obtained from GenBank. jModelTest v2.1.7 software was used to determine the nucleotide substitution model that was a best fit for each alignment based on the Akaike information criterion corrected for small sample sizes (Posada, 2008). Maximum likelihood (ML) and Bayesian inference (BI) were used to estimate phylogenies for the D2-D3 and COI regions. For ML, 250 bootstraps were used and the general time reversible model with allowance for a gamma distribution of rate variation (GTR + Γ) in RaxML v8 (Stamatakis, 2014). Inferred phylogenies by BI (MrBayes v3.2.6, Ronquist et al., 2012), used the general time reversible model with allowance for a gamma distribution of rate variation and a proportion of invariant sites (GTR + Γ + I) for LSU, and GTR + Γ for COI. Two independent metropolis-coupled Markov chain Monte Carlo (MCMCMC) searches for 2 million generations, sampled every 2,000 steps were used for both the D2-D3 and COI regions. Convergence was assessed in Tracer v1.5, using a burn in of 20%, and by examining the average standard deviation of split frequencies among parallel chains. A consensus tree was calculated for each region from the posterior distribution of 1,600 phylogenies. *Hoplolaimus seinhorsti* and *Hoplolaimus magnistylus* were used as outgroups for D2-D3 and COI, respectively, for the ML and Bayesian analyses (Miller et al., 2010).

## Results

### Morphological and morphometric identification

**Figure 1:**
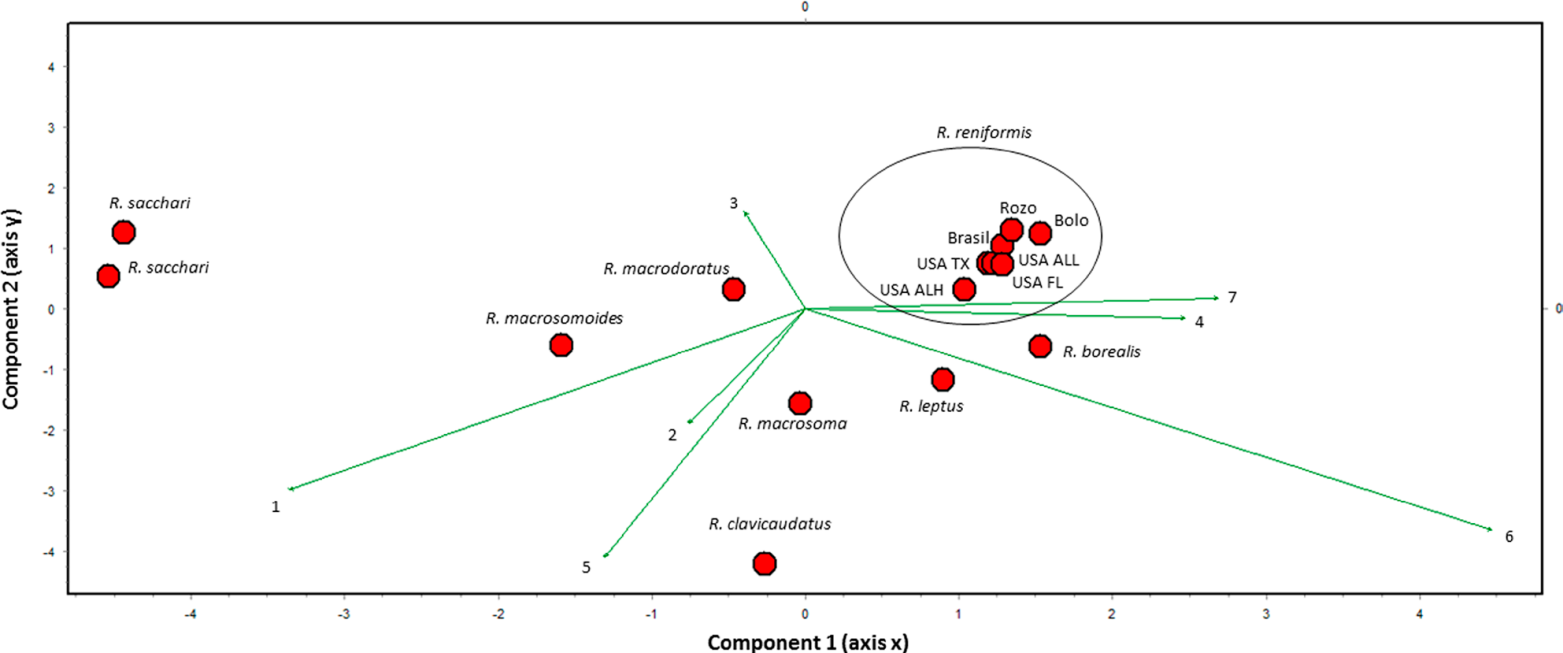
Biplot for Colombian and Brazilian populations of *Rotylenchulus reniformis* associated with banana and other species of the genus. The two first axes of a principal components analysis (PCA) are shown.

**Figure 2:**
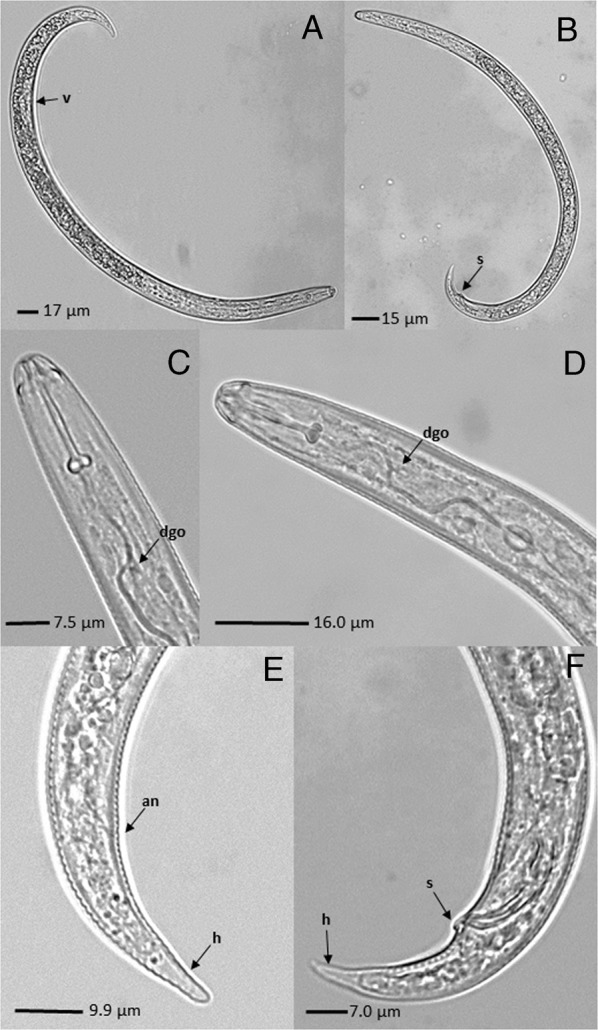
*Rotylenchulus reniformis.* (A) immature female; (B) male; (C and D) anterior region of body; (E) posterior region of immature female; and (F) posterior region of male. V = vulva; s = spicule; dgo = dorsal esophageal gland orifice; an = anus; h = tail hyaline.

The Colombian and Brazilian populations analyzed in this study were identified morphologically and morphometrically as *R. reniformis* (Table 1 and Fig. 1). Diagnostic characters and morphological characteristics for populations assessed in this study closely resembled those of type and topotype populations (Table 1 and Fig. 2A–F). Multivariate analysis showed that Colombian and Brazilian populations grouped closely with *R. reniformis* reference populations (from USA) but disparate to other valid species of *Rotylenchulus*, including *R. borealis*, *R. clavicaudatus*, *R. leptus*, *R. macrodoratus*, *R. macrosoma*, *R. macrosomoides*, and *R. sacchari* (according with measurements reported by Van den Berg et al., 2003, 2015; Agudelo et al., 2005). The Principal Components 1 and 2 had eigenvalues greater than or equal to 1 and explained 84% of variance. The first three principal components explained 94.6% of the variation recorded. The main influencing morphological/morphometric characters were L, a and stylet (PC1) and in PC2, c’, b, and V (Table 2).

**Table 2.** Correlations between the seven principal components and the morphometric parameters for immature females in *Rotylenchulus* spp.

Vector	Diagnostic character	1	2	3	4	5	6	7
1	L	**−** *0.50*	−0.08	0.01	0.36	−0.21	0.64	0.40
2	a	**−** *0.45*	−0.28	0.26	−0.06	−0.58	−0.56	0.03
3	b	−0.31	−*0.47*	0.28	−0.61	0.42	0.23	−0.09
4	c	−0.43	0.32	−0.36	−0.15	−0.18	0.18	−0.71
5	c’	0.21	**−** *0.59*	0.15	0.53	−0.01	0.13	−0.54
6	V	0.08	*0.46*	0.83	0.02	−0.11	0.19	−0.2
7	Stylet	−*0.47*	0.18	0.09	0.44	0.63	−0.37	−0.03

Note: Key diagnostics for discriminating Rotylenchulus species are denoted in italic.

### Molecular identification

Consensus sequences of the D2-D3 expansion region obtained for Colombian populations had a strong affinity (99% similarity) with a number of *R. reniformis* reference sequences (KP054126, KT003743, KP054077, KP054088, KT003744, KF999977, KF999978, and DQ328713). Similarly, COI sequences also had a strong affinity (99% similarity) with *R. reniformis* reference sequences (KT003727, KT003728, KT003729, KT003730, and KT003731). All sequences obtained in this study were deposited in NCBI under accession numbers MK879441-MK879450 (D2-D3) and MK908051-MK908060 (COI).

**Figure 3:**
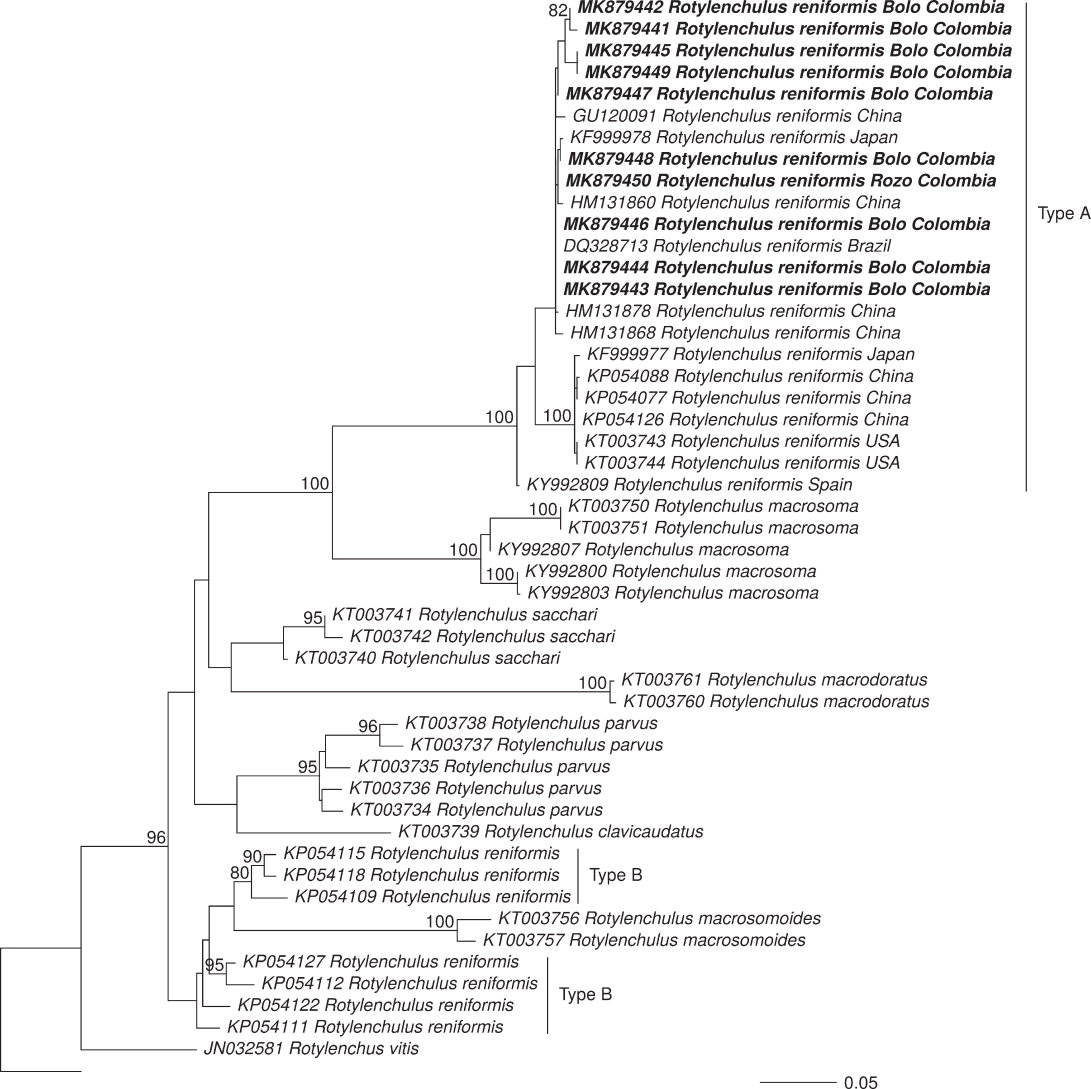
Maximum likelihood phylogenetic tree of *Rotylenchulus* based on D2-D3 expansion segment of 28 S ribosomal DNA and 250 bootstraps. The outgroup (*Hoplolaimus seinhorsti*) is shown in gray font; the sequences that were obtained in this study appear in bold typeface. Values at the nodes represent the posterior probability. The scale represents the number of substitutions per site.

**Figure 4:**
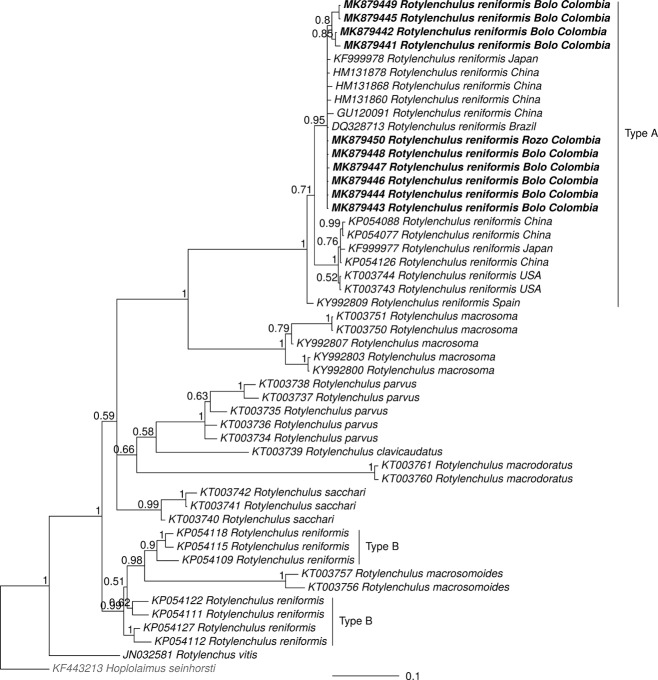
Bayesian phylogenetic tree of *Rotylenchulus* based on D2-D3 expansion segment of 28 S ribosomal DNA. The phylogeny is a consensus tree from a posterior distribution of 1,600 trees that were inferred in MrBayes. The outgroup (*Hoplolaimus seinhorsti*) is shown in gray font; the sequences that were obtained in this study appear in bold typeface. Values at the nodes represent the posterior probability. The scale represents the number of substitutions per site.

ML (Fig. 3) and Bayesian (Fig. 4) D2-D3 phylogenies clustered, with strong support, Colombian sequences of *R. reniformis* from this study with those of *R. reniformis* Type A. Irrespective of analytical method used (ML or BI), Colombian sequences of *R. reniformis* grouped with *R. reniformis* reference sequences associated with different host plants and geographic origin (KFF999978 of *Podocarpus macrophyllus* from Japan, HM131878, HM131868, HM131860, and GU120091 from China, and DQ328713 from Brazil) (Figs. 3-4).

**Figure 5:**
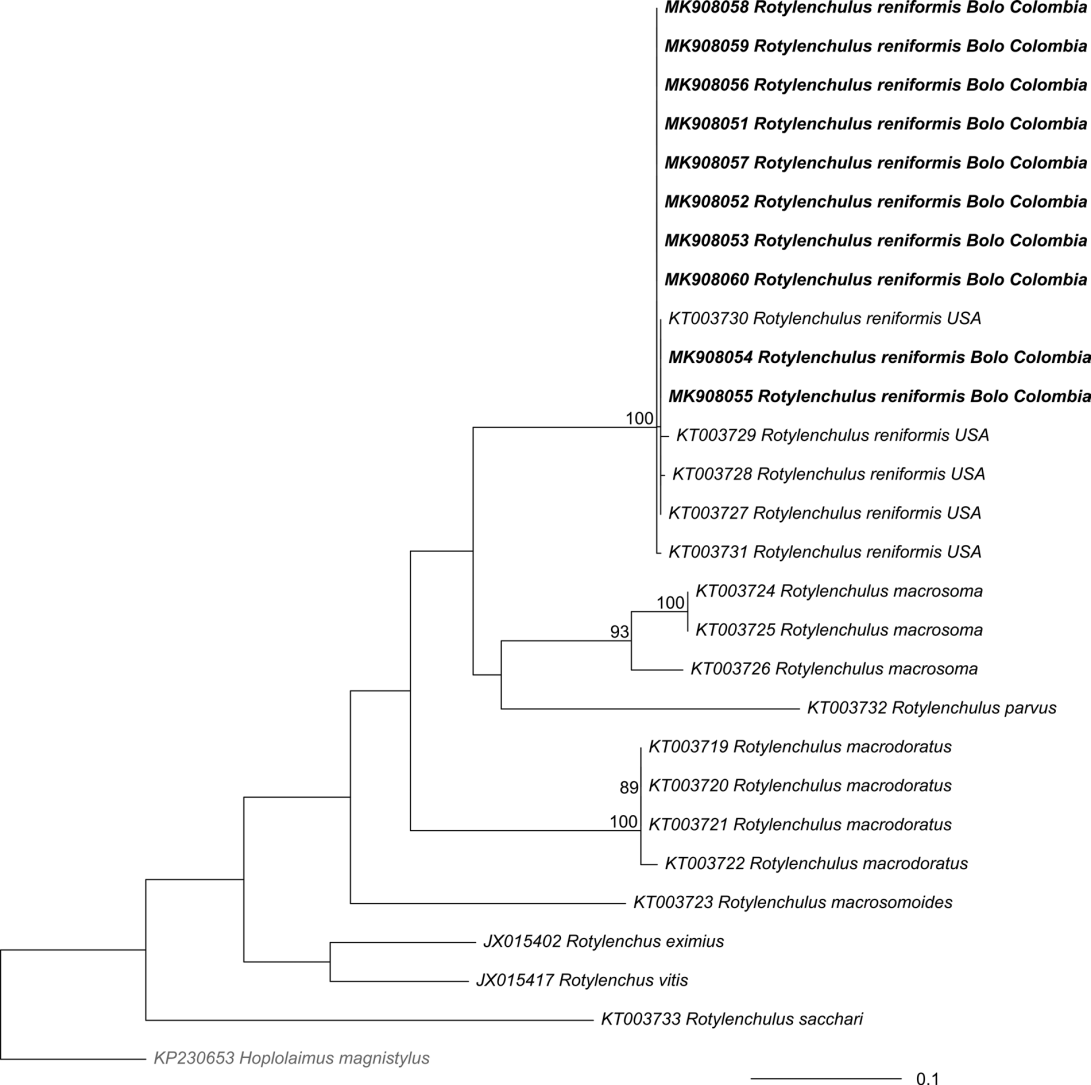
Maximum likelihood phylogenetic tree of *Rotylenchulus* based on mitochondrial cytochrome oxidase subunit I (COI) and 250 bootstraps. The outgroup (*Hoplolaimus magnystilus*) is shown in gray font; the sequences that were obtained in this study appear in bold typeface. Values at the nodes represent the posterior probability. The scale represents the number of substitutions per site.

**Figure 6:**
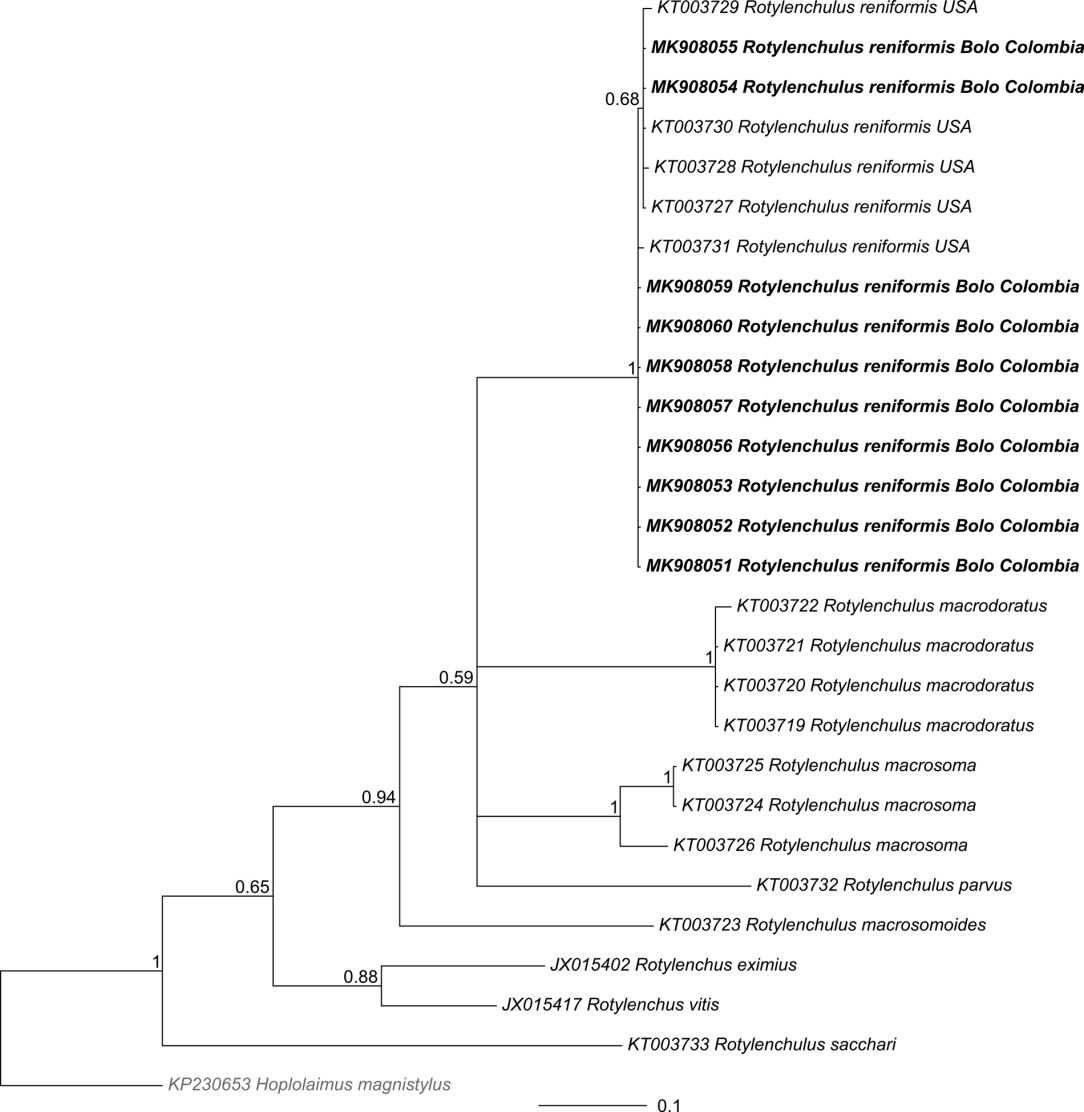
Bayesian phylogenetic tree of *Rotylenchulus* based on mitochondrial cytochrome oxydase subunit I (COI). The phylogeny is a consensus tree from a posterior distribution of 1,600 trees that were inferred in MrBayes. The outgroup (*Hoplolaimus magnystilus*) is shown in gray font; the sequences that were obtained in this study appear in bold typeface. Values at the nodes represent the posterior probability. The scale represents the number of substitutions per site.

All Colombian *R. reniformis* COI sequences clustered together with other *R. reniformis* populations (Figs. 5-6, BS = 100%, PP = 1). In both phylogenies, Colombian populations grouped with *R. reniformis* associated with different host plants and geographic origin (KT003727 from Florida, USA, KT003728 of cotton from Arkansas, USA, KT003729 of *Sansevieria* sp. From Florida, USA, KT003730 of *Euphorbia* sp. From Florida, USA and KT003731 of *Yucca elephantipes* from Florida, USA) (Figs. 5-6).

## Discussion

Nematodes associated with plantain and banana analyzed in the present study were identified as *R. reniformis* by morphological, morphometric, and molecular methods. With regard to the morphometric analysis, measurements closely resembled those reported for type and topotype populations of *R. reniformis* and published dichotomous keys (Linford and Oliveira, 1940; Dasgupta et al., 1968; Robinson et al., 1997; Agudelo et al., 2005; Palomares-Rius et al., 2018). However, differences were noted for some diagnostic characters between the studied and reference populations suggesting intraspecific variation. Such variation is reported to be driven by temperature, nutrients and growth conditions of the host plant (Evans and Fisher, 1970; Nakasono, 2004; Nyaku et al., 2013, 2016).

Morphological identification of *Rotyl Peter Ramley and Roy enchulus* species is considered problematic due to a high degree of intraspecific variation (Dasgupta et al., 1968; Germani, 1978; Robinson et al., 1997). Notwithstanding, the intraspecific variation encountered in this study, key discriminatory diagnostic characters (L, stylet, b, c, c’, and V) were identified through the use of multivariate analysis that supported robust identification of *R. reniformis* and separated the species from the other valid *Rotylenchulus* species (Linford and Oliveira, 1940; Dasgupta et al., 1968; Robinson et al., 1997; Van den Berg et al., 2015).

*Rotylenchulus borealis* is a species reported in the banana crops of Cameroon, Kenya, South Africa, and Rwanda. However, literature revised show marked morphometric differences between *R. borealis* and *R. reniformis* populations analyzed in this study (Van den Berg et al., 2003). With regard to measurements of immature females, the principal differences between both species were body length (L), dorsal gland orifice (DGO), pharynx length, excretory pore, lip region height, and tail length, with higher values attributed to *R. borealis* (Van den Berg et al., 2003; Gaidashova et al., 2004).

Assessment of the D2-D3 and COI regions of the nematodes in our study had a strong affinity to previously published sequences attributed to *R. reniformis.* This was consistent with the morphometric and morphological data generated in this study. Tree topologies generated by ML and BI methods were similar. Our present study confirmed the results of Van den Berg et al. (2015) who found two distinct types of D2-D3 28 S rRNA in the *R. reniformis* genome. Type A, including all the studied Colombian populations, formed a well-supported group with Brazil, China, Japan, Spain, and USA populations and Type B which was disparate from Type A. However, the relation between type and pathogenicity or virulence is unknown. *R. reniformis* Type A has been reported associated with a range of economically important crops, including cotton (KY992808) (Van den Berg et al., 2015; Palomares-Rius et al., 2018).

Based on PCA, the single Brazilian population studied grouped with Colombian populations identified morphometrically and molecularly as *R. reniformis*. This species has previously been reported associated with different crops in Brazil such as: *Lycopersicum esculentum* Mill., *Gossypium hirsutum* L., *Carica papaya* L., *Glycine max* (L.) Merril., *Phaseolus vulgaris* L., *Passiflora edulis* Sims., and *Ananas comosus* (L.) Merr. (Soares et al., 2003). It has also been reported to be associated with banana production in the Brazilian states of Bahia, Ceará, Paraíba, Rio de Janeiro, and Espirito Santo (Costa Manso et al., 1994).

The identification of *R. reniformis* in plantain and banana crops of Colombia and Brazil in the present study is consistent with previous reports of this nematode with *Musa* spp. from across the world (Fargette and Quénéhervé, 1988; Ngoc Chau et al., 1997; Khan and Hasan, 2010; Kamira et al., 2013; Daneel et al., 2015). This is the first report of *R. reniformis* in plantain and banana for Colombia through integrative taxonomy, contributing to the knowledge of the parasitic nematode community of this country, and is essential information for the future design of integrated management programs for *R. reniformis* associated with *Musa* spp. (Robinson et al., 1997; Crozzoli et al., 2004).
